# Supplementary material for: Anti-Hermitian photodetector facilitating efficient subwavelength photon sorting
Source: Nat Commun. 2018 Jan 22;9:316. doi: 10.1038/s41467-017-02496-y (PMC5778063; doi:10.1038/s41467-017-02496-y)
Supplement: Supplementary file 1 — Supplementary Information [file 41467_2017_2496_MOESM1_ESM.pdf]

Supplementary Note 1. Theoretical analysis of anti-Hermitian coupled system for the proposed photon-sorter

To model anti-Hermitian coupling of open quantum system, we develop a temporal coupled mode theory (CMT) for the proposed semiconductor nanobeam array. We start by analyzing a single-sized nanobeam array that features a deep-subwavelength spacing. Such an array couples to incident light through a single radiation channel as it only supports 0th diffraction order. In this regime is essentially behaves as a metamaterial and the CMT takes a simple form[1]:

$$\frac{dc}{dt} = (i\omega_1 - \gamma_\mathrm{a} - \gamma_\mathrm{r})\boldsymbol{c} + \kappa\, S_+ \quad (1.1)$$

$$S_- = B\, S_+ + d\, \boldsymbol{c} \quad (1.2)$$

where, $\boldsymbol{c}$, S_+ and S_- denote the resonant amplitude, and the amplitudes of the incoming and outgoing waves, respectively, with the normalization chosen such that $|c|^2$, $|S_+|^2$, and $|S_-|^2$ correspond to the energy inside the resonator and the power in the incoming and outgoing waves. ω_0, γ_a and γ_r represent the resonance frequency, loss rate by absorption and loss rate by re-radiation. Also, κ, B and d respectively indicate the coupling strength between the resonator and incident light, direct reflection and re-radiation from the resonator, respectively. As a next step, we apply energy conservation:

$$\frac{d|\boldsymbol{c}|^2}{dt} = |S_+|^2 - 2\gamma_\mathrm{a}|c|^2 - |S_-|^2 \quad (2)$$

where,

$$\frac{d|\boldsymbol{c}|^2}{dt} = \boldsymbol{c}\,\frac{d\boldsymbol{c}^*}{dt} + \boldsymbol{c}^*\,\frac{dc}{dt}$$
$$= \boldsymbol{c}(-i\omega_1 - \gamma_\mathrm{a} - \gamma_\mathrm{r})\boldsymbol{c}^* + \boldsymbol{c}\kappa^* S_+{}^* + \boldsymbol{c}^*(i\omega_1 - \gamma_\mathrm{a} - \gamma_\mathrm{r})\boldsymbol{c} + \boldsymbol{c}^*\kappa S_+ \quad (2.1)$$

$$|S_+|^2 - 2\gamma_\mathrm{a}|c|^2 - |S_-|^2$$
$$= |S_+|^2 - 2\gamma_\mathrm{a}|c|^2 - |B|^2|S_+|^2 - |d|^2|\boldsymbol{c}|^2 - BS_+d^*\boldsymbol{c}^* - B^*S_+{}^*d\boldsymbol{c} \quad (2.2)$$

By solving (2), we derive the following simplified relation.

$|d|^2 = 2\gamma_r$ (3.1)

$|B|^2 = 1$ (3.2)

$Bd^* = -\kappa$ (3.3)

We have chosen the reference plane such that that B = -1, as resulting from the direct reflection from the metal substrate. Based on time-reversal constraints[1], we further obtain $\kappa = d = \sqrt{2\gamma_r}$. At steady state with a sinusoidal incoming wave of $S_+ = |S_+|e^{i\omega t}$ and $\boldsymbol{c} = |c|e^{i\omega t}$, we obtain a coupled mode equation of the following form:

$$i(\omega - \omega_1)\boldsymbol{c} + (\gamma_a + \gamma_r)\boldsymbol{c} = \kappa\, S_+ \quad (4)$$

$$S_- = -1 \cdot S_+ + \kappa\, \boldsymbol{c} \quad (5)$$

We can now write expressions for the spectral reflectivity properties of the beam-array. From (4) and (5), the reflectivity can be written as:

$$\left|\frac{S_-}{S_+}\right|^2 = \left| -1 + \frac{\kappa^2}{i(\omega-\omega_1) + (\gamma_a + \gamma_r)} \right|^2 \quad (6)$$

We can link the relevant parameters of the CMT to those obtained from experiment or electromagnetic simulations. The absorption/radiation losses as well as the coupling coefficients occurring in Eq.(6) can be linked to the full-width at half-maximum (FWHM) and depth of the of the reflection dip:

$$(\gamma_a + \gamma_r) = \frac{FWHM}{2} \quad (7.1)$$

$$\left|\frac{S_-}{S_+}\right|^2_{w=w1} = \min(R) \quad (7.2)$$

From the experiments or simulations we can thus extract values for γ_a, γ_r and the coupling coefficient κ. This analysis was performed for the single-sized nanobeam arrays, whose reflection spectrums are shown in Fig. 3. Good agreement was found between the CMT and the

simulated/measured reflection spectrums. With knowledge of these quantities, we can also calculate the predicted absorption $P_{\mathrm{abs}} = 2\,\gamma_{\mathrm{a}}\,|\boldsymbol{c}|^2$ for arrays of single-sized nanobeams. The spectral absorption properties of the wide and narrow beams are shown in Supplementary Figure 1a. The two beam sizes display resonantly enhanced absorption at wavelengths of 595 nm (blue) and 625 nm (red), respectively. The lineshape of each resonance is a simple Lorentzian.

In order to understand how the coupling between the narrow and wide beams can affect the absorption properties, one can develop a CMT for a system of two coupled resonances that can be accessed via a single optical channel (1). We can write a similar set of equation for a coupled system where the subscripts denote the properties of resonance 1 and 2:

$$\frac{d\boldsymbol{c_1}}{dt} = (i\omega_1 - \gamma_{\mathrm{a1}} - \gamma_{\mathrm{r1}})\boldsymbol{c_1} + (i\omega_{12} - \gamma_0)\boldsymbol{c_2} + \kappa_1\,S_+ \qquad (8.1)$$

$$\frac{d\boldsymbol{c_2}}{dt} = (i\omega_2 - \gamma_{\mathrm{a2}} - \gamma_{\mathrm{r2}})\boldsymbol{c_2} + (i\omega_{12} - \gamma_0)\boldsymbol{c_1} + \kappa_2\,S_+ \qquad (8.2)$$

$$S_- = -1 \cdot S_+ + \kappa_1\,\boldsymbol{c_1} + \kappa_2\,\boldsymbol{c_2} \qquad (8.3)$$

where w_{12} indicates the direct coupling term and γ_0 indicates the indirect coupling term between the interacting resonant systems. To evaluate the value of γ_0, we again use the energy conservation and this gives the following relations.

$$\frac{d}{dt}(|\boldsymbol{c_1}|^2 + |\boldsymbol{c_2}|^2) = |S_+|^2 - 2\gamma_{\mathrm{a1}}|\boldsymbol{c_1}|^2 - 2\gamma_{\mathrm{a2}}|\boldsymbol{c_2}|^2 - |S_-|^2 \qquad (9)$$

where,

$$\begin{aligned}\frac{d}{dt}(|\boldsymbol{c_1}|^2 + |\boldsymbol{c_2}|^2) &= \boldsymbol{c_1}\frac{d\boldsymbol{c^*}_1}{dt} + \boldsymbol{c^*}_1\frac{d\boldsymbol{c_1}}{dt} + \boldsymbol{c_2}\frac{d\boldsymbol{c^*}_2}{dt} + \boldsymbol{c^*}_2\frac{d\boldsymbol{c_2}}{dt} \\ &= -(2\gamma_{\mathrm{a1}} + 2\gamma_{\mathrm{r1}})|\boldsymbol{c_1}|^2 - (2\gamma_{\mathrm{a2}} + 2\gamma_{\mathrm{r2}})|\boldsymbol{c_2}|^2 - 2\boldsymbol{c_1}\boldsymbol{c_2}^*\gamma_0 \\ &\quad -2\boldsymbol{c_1}^*\boldsymbol{c_2}\gamma_0 + \boldsymbol{c_1}^*S_+\kappa_1 + \boldsymbol{c_2}^*S_+\kappa_2 + \boldsymbol{c_1}S_+^*\kappa_1^* + \boldsymbol{c_2}S_+^*\kappa_2^*\end{aligned} \qquad (9.1)$$

$$\begin{aligned}&|S_+|^2 - 2\gamma_{\mathrm{a1}}|\boldsymbol{c_1}|^2 - 2\gamma_{\mathrm{a2}}|\boldsymbol{c_2}|^2 - |S_-|^2 \\ &= (1 - |B|^2)|S_+|^2 - (2\gamma_{\mathrm{a1}} + |d_1|^2)|\boldsymbol{c_1}|^2 - (2\gamma_{\mathrm{a2}} + |d_2|^2)|\boldsymbol{c_2}|^2 - \boldsymbol{c_1}\boldsymbol{c_2}^*\kappa_1\kappa_2^* \\ &\quad -\boldsymbol{c_1}^*\boldsymbol{c_2}\kappa_1^*\kappa_2 - \boldsymbol{c_1}^*S_+B\kappa_1^* - \boldsymbol{c_2}^*S_+B\kappa_2^* - \boldsymbol{c_1}S_+^*B^*\kappa_1 - \boldsymbol{c_2}S_+^*B^*\kappa_2\end{aligned} \qquad (9.2)$$

By solving (9), we can obtain the indirect coupling strength γ_0 as a function of γ_{r1} and γ_{r2}:

$$\gamma_0 = \sqrt{\gamma_{\mathrm{r1}}\,\gamma_{\mathrm{r2}}} \qquad (10)$$

The CMT for the coupled system can be written in a simple matrix form:

$$\frac{\partial}{\partial t}\begin{bmatrix} \boldsymbol{c_1} \\ \boldsymbol{c_2} \end{bmatrix} = i\begin{bmatrix} \omega_1 + i(\gamma_{\mathrm{a1}} + \gamma_{\mathrm{r1}}) & \omega_{12} + i\gamma_0 \\ \omega_{12} + i\gamma_0 & \omega_2 + i(\gamma_{\mathrm{a2}} + \gamma_{\mathrm{r2}}) \end{bmatrix}\begin{bmatrix} \boldsymbol{c_1} \\ \boldsymbol{c_2} \end{bmatrix} + \begin{bmatrix} \kappa_1 \\ \kappa_2 \end{bmatrix} S_+ \qquad (11)$$

In steady state with a sinusoidal incoming wave, we obtain the following expression for the amplitudes $\boldsymbol{c_1}$ and $\boldsymbol{c_2}$:

$$i\begin{bmatrix} (\omega - \omega_1) - i(\gamma_{\mathrm{a1}} + \gamma_{\mathrm{r1}}) & -\omega_{12} - i\gamma_0 \\ -\omega_{12} - i\gamma_0 & (\omega - \omega_2) - i(\gamma_{\mathrm{a2}} + \gamma_{\mathrm{r2}}) \end{bmatrix}\begin{bmatrix} \boldsymbol{c_1} \\ \boldsymbol{c_2} \end{bmatrix} = \begin{bmatrix} \kappa_1 \\ \kappa_2 \end{bmatrix} S_+ \qquad (12)$$

Similar to the Supplementary Figure 1a, we can plot the expected absorption for a system of coupled resonantors for different types of coupling. Supplementary Figure 1b shows the spectral absorption of the system for a case that an anti-Hermitian coupling is achieved. Such a coupling is realized by suppressing the near-field interaction, i.e. setting the term w_{12} to a zero value, and by maximizing the far-field interaction term γ_0 by choosing the right solution of γ_{r1} and γ_{r2} from the single-sized nanobeam array, that produces a π phase difference in re-radiation. Physically, this condition enforces that the systems interacts only indirectly in the far-field without near-field coupling. Such coherent destructive interference in the far-field regime results in a decreased radiation and thus an increased Q_{rad}. This results in a narrowing of the resonance. The lineshape of the resonances becomes asymmetric as the relative amplitude of the interfering scattered fields from the beams varies across the two resonances. Intriguingly, when one beam is on resonance, its amplitude is increased at the expensive of the other beam. This is consistent with the powerflow images in Fig.1 d,e that show an enhanced absorption cross section for the beam that is on resonance. On the contrary, when we simply activate the direct, near-field interaction by setting a non-zero value for w_{12}, a mode splitting is realized between the two resonances, as seen in Supplementary Figure 1c. In this case, the absorption peaks are spectrally shifted and thus, the system cannot be utilized as a photon sorter.

Based on the above analysis, we now more precisely fit the parameters of the theory to the simulation results as seen in Fig 3-a, c and e. To improve accuracy, we build a more realistic model by incorporating the spectral changes in the intrinsic absorption of silicon and a weak near-field interaction between the two resonances. Specifically, we initially fit the resonance wavelength and leakage rate from the spectrums shown in Fig 3-a. Using these values, we subsequently calculate the coupling constant, γ_0, to plot Fig 3-b and c. As seen in the figure, the theory fits well to the simulated absorption spectrum, which implies that the designed device operates with an effective anti-Hermitian coupling.

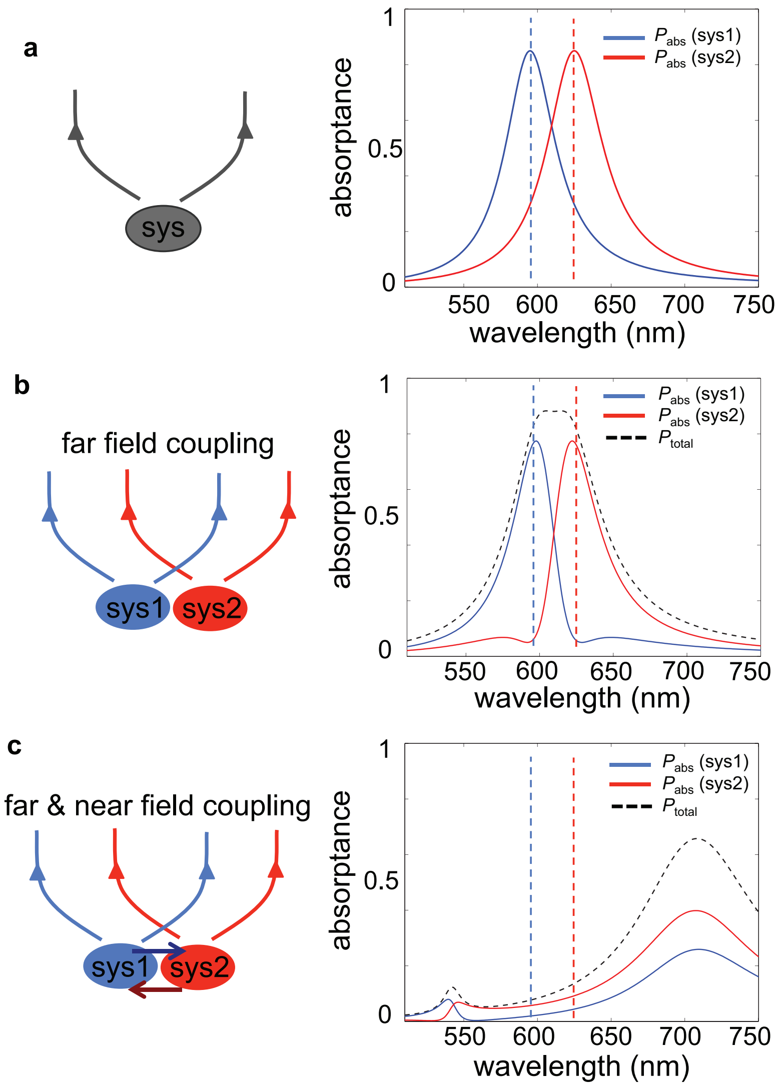

Supplementary Figure 1. Absorption spectrum from three representative systems developed by coupled mode theory. (a) Absorption spectrum for a system with just narrow and just wide nanobeams as coupled to a single radiation channel. **(b)** Absorption spectrum for a metafilm with interlaced narrow and wide beams with an anti-Hermitian coupling between them. The wire

widths are chosen to be the same as in panel (a). **(c)** Absorption spectrum from a metafilm with interlaced narrow and wide beams with that interact via both far-field and near-field coupling. The near-field coupling causes the resonant modes to spectrally split.

Supplementary Note 2. Achieving anti-reflection via anti-Hermitian coupling

In this section, we analyze how anti-Hermitian coupling in our proposed structure plays a role suppressing reflection and enhancing light absorption. This is desirable as it naturally increases the quantum yield of the photodetector. We start by investigating an array composed of single sized nanobeams as seen in Supplementary Figure 2a and b. In Supplementary Figure 2a, the period of the beams is optimized and thus the absorption in silicon at a wavelength of 595 nm is close to unity as seen in Supplementary Figure 2f. Such strong absorption results from a cancellation of the reflected light between resonant nanobeams and metal surface. To visualize this, we decompose the total field into the scattered and the incident field, i.e. $\boldsymbol{E}_{\text{tot}} = \boldsymbol{E}_{\text{scat}} + \boldsymbol{E}_{\text{inc}}$, and display the phase of the scattered field as seen in Supplementary Figure 2a. From this figure, it is clear that the scattered field (i.e. the reflected light), near the surface of the resonant nanobeam and near the metal surface are π out of phase. This results in a destructive interference between these two scattered fields and a suppression of reflected light. In this case, the amplitudes of the scattered fields are similar and near-critical coupling is achieved.

On the other hand, when the additional nanobeams are placed in the array, we lose the metal regions that reflected the light with a π phase difference to cause destructive interference of the outgoing, reflected wave. It can be seen that the reflected light from each nanobeam is now in phase, causing an effective constructively interference. This results in a high reflectivity. As a result, the light absorption in in the nanobeams seen in Supplementary Figure 2g is significantly lower than seen in Supplementary Figure 2f. In this example, most of the light flows through the resonant nanobeams back into the reflected light channel and the system in an over-coupled state.

Next, we explore what happens as we gradually increase the width of the additionally inserted nanobeams from 60 nm to 80 nm to form a metafilm with two distinct beam sizes. Three representative examples with the width of 65 nm, 70 nm and 80 nm are shown in Supplementary Figure 2c, d and e. We show both the spectral absorption in the two beams as well as maps of the scattered field at the wavelength right between the resonances of the two differently-sized beams. These illustrate the importance of controlling the relative phase of the scattered light emerging from the different beam sizes. For small differences in beam width, the scattered fields from the beams are not perfectly out of phase and the reflected wave is not perfectly canceled. For this reason, near-unity absorption is not achievable. At the width of 80 nm in Fig 3-e, the phase

difference of the scattered light from narrower and wider beam is now close to π at the wavelength of 595 nm. The resulting cancellation of the scattered fields from the nanobeams suppresses the formation of a reflection wave, and near-critical coupling is achieved with a more broadband absorption than for the single nanobeam example shown in Supplementary Figure 2a.

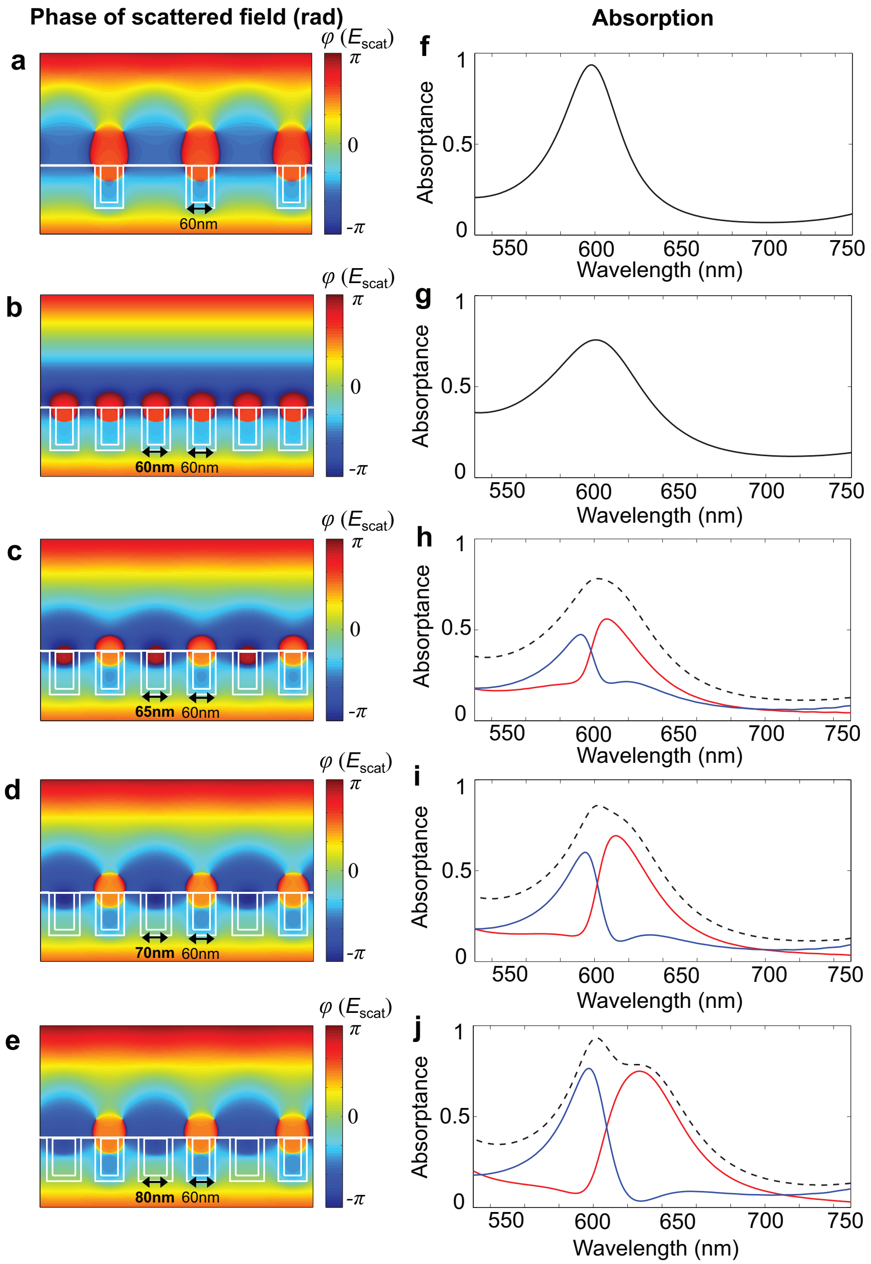

Supplementary Figure 2. Achieving anti-reflection through destructive interference of scattered light. (a-e) Images showing the phase of the simulated scattered field from different nanobeam arrays upon illumination at a wavelength of 595 nm. **(f-j)** Absorption spectrum of the

corresponding nanostructures. Panels a and f shows that the scatted fields from a single-sized nanobeam and the metal surface can feature an approximate phase difference of π. This leads to destructive interference and a cancelation of the reflected light. Panel b and g show how the subtle destructive interference condition is disrupted when the density of the beams is increased. The field maps in panels c,d,e, and the spectrums shown in panels h,i, and j shows that a new destructive interference condition can be achieved that lead to the cancelation of the reflected light and a strong absorption. Here, the cancelation results from a destructive interference of the light scattered from the narrow and wide nanobeams in the interlaced metafilm device. Panel j shows that more broadband, near-unity absorption as well as effective photon sorting can be achieved by having two nanobeam sizes present.

Supplementary Note 3. Map of light absorption with the display of mode splitting at the very deep subwavelength beam array

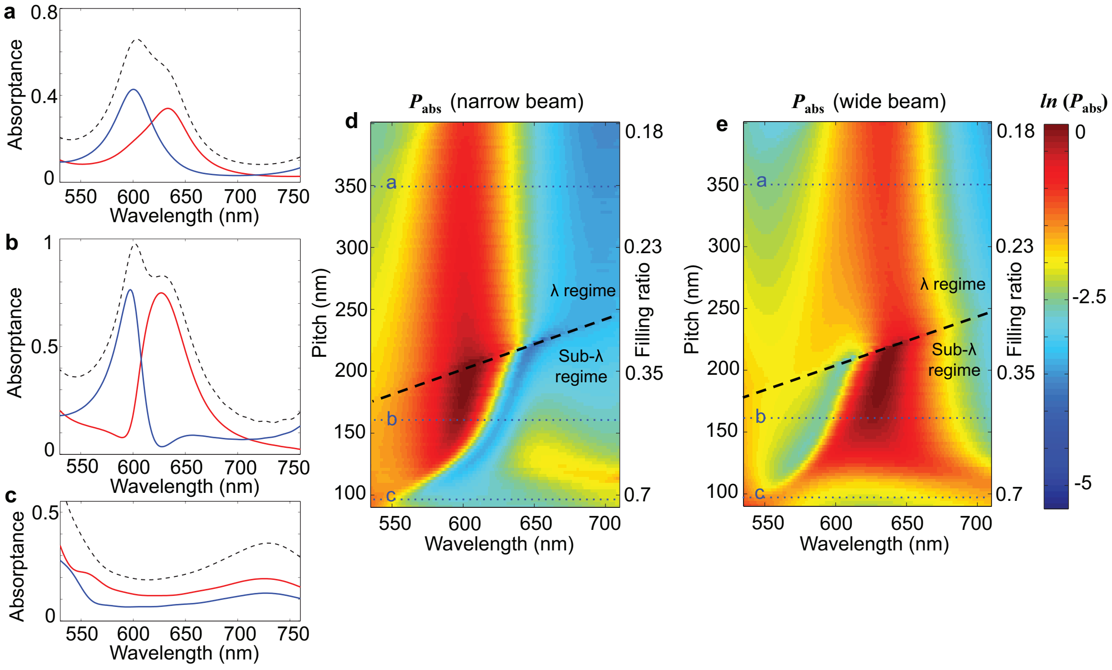

Supplementary Figure 3. Map of light absorption with the spectral crosssections at three pitch points. (a) Absorption spectrum of the nanobeam array with the pitch size of 350nm. **(b)** Absorption spectrum of subwavelength-scaled nanobeam array with the pitch size of 160nm. **(c)** Absorption spectrum of very deep subwavelength with the pitch size of 95nm. **(d, e)** Absorption map with respect to the wavelength and the pitch.

Supplementary Note 4. Sorting light across a broadband spectrum for solar energy harvesting

The proposed photodetector not only is capable of sorting photons into narrowband wavelength channels, but also can effectively sort more spectrally separated and broadband channels. To prove this, we design a multi-sized Si-based nanobeam-array with width of 50 nm and 100 nm. This represents a larger size contrast than in the previously discussed designs. The height of all the nanobeams is fixed as 120 nm. We first run full-field simulations and analyze the performance of the device by evaluating reflection and absorption as seen in Supplementary Figure 4a and c. From the figures, it is observed that there are spectrally separated two reflection dips and absorption peaks, respectively. We also experimentally demonstrate broadband photon sorting by measuring the reflectivity and extracted photocurrent of the fabricated devices. As seen in Supplementary Figure 4b and d, we again achieve reflection dips and photocurrent peaks experimentally whose wavelength matches well with the simulated expectations.

Such devices can be used in a variety of applications that require broadband photon sorting, for instance, next generation solar energy applications. In this regard, it would be very interesting to speculate on possible designs where the beams are made from different bandgap materials to maximize performance in a similar fashion as multi-junction solar cells can outperform single junction cells. In this case a higher performance could be achievable within a single layer device. In this way, we not only achieve efficient light absorption, but also harness the bandgap of materials that contributes to an increased open circuit voltage. To explore this opportunity, we analyze a design consisting of interlaced nanobeams made from silicon and perovskite. After an optimization step we find that 50-nm-wide and 90-nm-hight perovskite beams interlaced with 90-nm-wide and 130-nm-high Si beams provides good results. Since the perovskite nanobeams are smaller than the Si nanobeam, they resonate and absorb light at a shorter wavelength. From these wires we can extract current with a relatively higher open circuit voltage. On the other hand, silicon has lower bandgap than perovskite and produces photocurrent from longer wavelength photons at a lower open circuit voltage. Supplementary Figure 5 shows the simulated absorption spectrum for each material in our proof-of-concept device. Whereas the strongest absorption in the perovskite near a wavelength of 550 nm, the silicon absorbs dominantly near a wavelength of 650 nm.

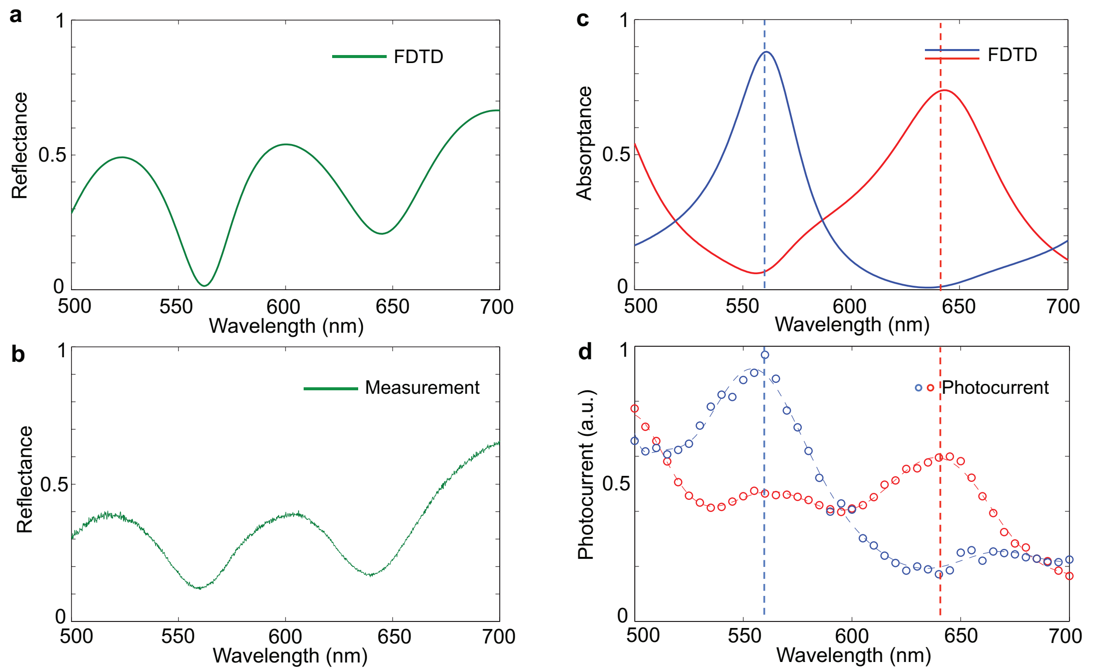

Supplementary Figure 4. Demonstration of broadband, spectrally sorted absorption of light. (a) Simulated reflection spectrum of interlaced Si nanobeams with the width of 50 nm and 100 nm. **(b)** Experimentally measured reflection spectrum from the fabricated sample. **(c)** Simulated absorption spectrum showing the fraction of absorbed light in the narrow beams (red) and in wide beams (blue). **(d)** Experimentally measured photocurrent spectrum extracted from the narrow beams (blue circles) and from wide beams (red circles).

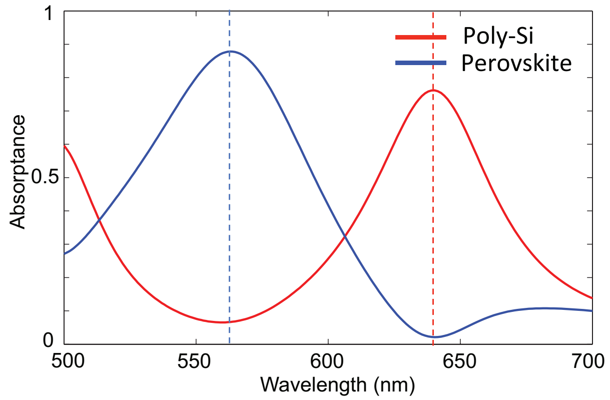

Supplementary Figure 5. Simulated absorption spectrums for an ultrathin tandem solar cell with lateral spectral splitting in perovskite and poly-silicon nanobeams. Blue line indicates the absorption in perovskite nanobeams that have higher bandgap than the bandgap of silicon. Red line indicates the absorption in silicon nanobeams that feature a small bandgap, which are suitable for harvesting light at long wavelengths.

Supplementary Note 5. SEM image of a measured device and the description of a proposed single device.

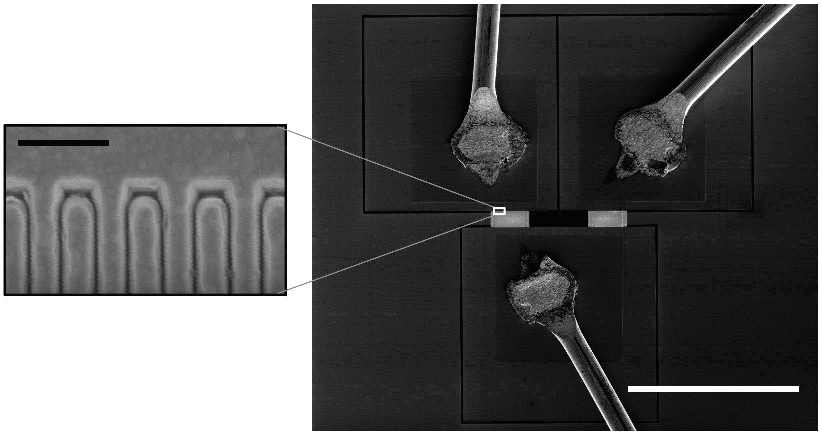

Supplementary Figure 6. SEM image of a measured device. Two nominally identical nanowire devices are fabricated on a single substratee and electrically connected via wirebonds. For one device, the electrical connections are made to the wide nanobeams and in the other device the narrow beams are connected. This enables photocurrent extraction from either the wide or narrow nanobeam array. During a measurement, three electrodes (including common cathode) are biased at 2V to facilitate effective current extraction. Scale bar, 500nm (left) and 200μm (right).

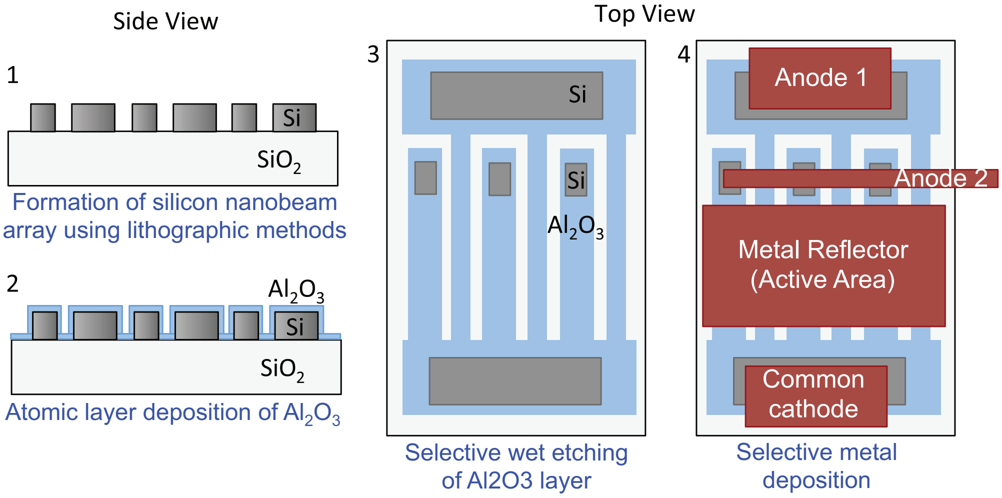

Supplementary Figure 7. Description of a proposed single device. A single device which extracts photocurrent from different-sized nanobeams simultaneously is described and proposed as seen in the figure above. The Al_2O_3 layer (blue) is selectively etched, and metallic lectrodes (red) are deposited on locally exposed silicon features (grey). Anode 1 and anode 2 can extract photocurrent from the wide and narrow nanobeams separately.

Supplementary Note 6. Impact of fabrication-related imperfections on the device performance

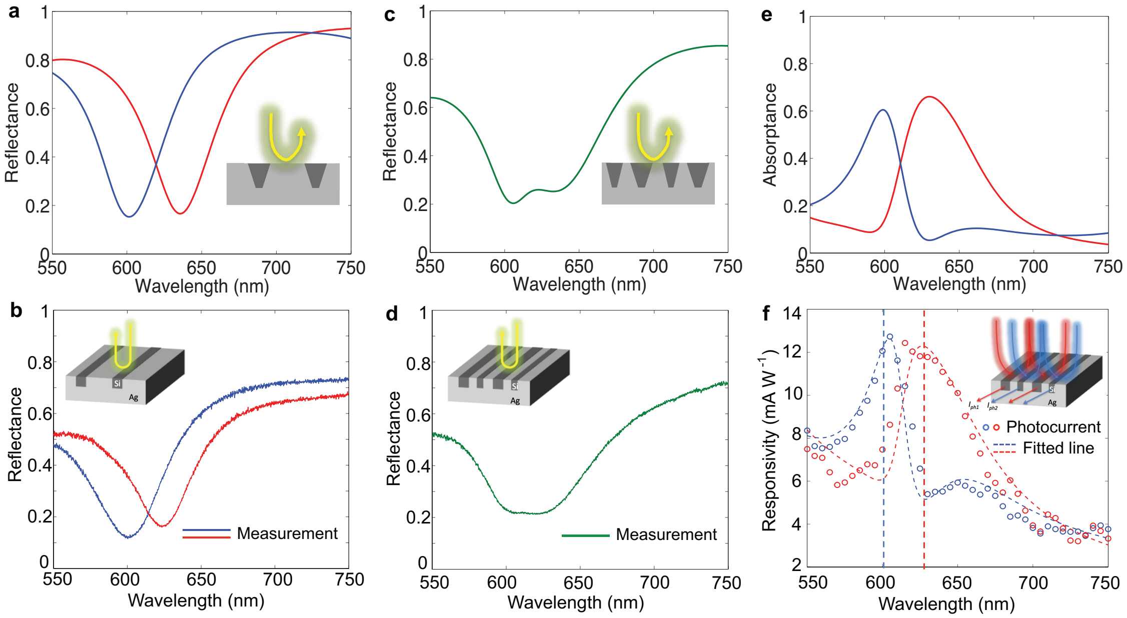

Supplementary Figure 8. Simulation of a real device in which the nanobeams display non-vertical sidewalls size variations. (a) Simulated reflectance spectrums of single-sized nanobeam arrays obtained by FDTD simulations of the fabricated device structure with dimensions and sidewall slope extracted from electron microscopy images for beam widths of 60 nm (blue) and 80 nm (red), respectively. (**b**) Measured reflectance spectrums as repeated from Fig. 3b. **(c)** Simulated reflection spectrums of the fabricated device in which the two types of the nanobeams are interlaced. (**d**) Measured reflectance spectrums as repeated from Fig. 3d **(e)** Simulated absorption spectrum of the fabricated device with a the multi-sized nanobeam array. Fraction of the absorption is shown in blue for narrow and red for wide nanobeam array. **(d**) Measured responsivity spectrums as repeated from Fig. 3f.

It is noted that due to the non-vertical side walls and the slight deviations of nanobeam sizes, the experimental measurements can not achieve near-unity absorption. We model this by extracting the dimensions of this device from electron microscopy images that show a nanobeam array with non-vertical side walls and some variation in the beam widths. The width of non-vertical nanobeam for this device was found to be 80 nm/100 nm on the top and 50 nm/70 nm at the bottom for the narrow/wide beams. The width variation of about 6 nm were observed and assumed in this simulation.

Supplementary References

1. Fan, S., Suh, W. & Joannopoulos, J. D., Temporal coupled mode theory for the Fano resonance in optical resonators. *J. Opt. Soc. Am. A.* **20,** 569–572 (2003).
